# Nanoporous Silicon as a Green, High-Tech Educational Tool

**DOI:** 10.3390/nano11020553

**Published:** 2021-02-23

**Authors:** Jeffery L. Coffer, Leigh T. Canham

**Affiliations:** 1 Department of Chemistry and Biochemistry, Texas Christian University, Fort Worth, TX 76129, USA; 2 School of Physics and Astronomy, University of Birmingham, Edgbaston, Birmingham B15 2TT, UK

**Keywords:** nanoporous, silicon, green chemistry, sustainability, education, entrepreneurship

## Abstract

Pedagogical tools are needed that link multidisciplinary nanoscience and technology (NST) to multiple state-of-the-art applications, including those requiring new fabrication routes relying on green synthesis. These can both educate and motivate the next generation of entrepreneurial NST scientists to create innovative products whilst protecting the environment and resources. Nanoporous silicon shows promise as such a tool as it can be fabricated from plants and waste materials, but also embodies many key educational concepts and key industrial uses identified for NST. Specific mechanical, thermal, and optical properties become highly tunable through nanoporosity. We also describe exceptional properties for nanostructured silicon like medical biodegradability and efficient light emission that open up new functionality for this semiconductor. Examples of prior lecture courses and potential laboratory projects are provided, based on the author’s experiences in academic chemistry and physics departments in the USA and UK, together with industrial R&D in the medical, food, and consumer-care sectors. Nanoporous silicon-based lessons that engage students in the basics of entrepreneurship can also readily be identified, including idea generation, intellectual property, and clinical translation of nanomaterial products.

## 1. Introduction

### 1.1. Nanomaterial Education

Materials, useful matter, are used extensively and a few have enormous influence on how we live. Nanoscale science and technology (NST), a major growth area, is enabling us to both modify existing materials and invent new ones for a range of functions. Nanostructuring a bulk material into a “nanomaterial” can radically change its properties. The dominant semiconductor silicon is a classic example to be discussed here. 

There is a growing convergence between learning and utilizing more how natural living systems operate at the nanoscale, whilst protecting our environment and resources [1,2,3,4]. NST is, in this regard, intrinsically multidisciplinary, often utilizing knowledge from chemistry, physics, engineering, biology, medicine, and ecology, to name a few disciplines [5]. There is also a continuing need to update university- and school-level curricula with contemporary scientific topics, and nanoscience and nanomaterials are no exception [6,7,8,9]. If we take the chemical synthesis and properties of nanoparticles as an example, then many papers in journals dedicated to teaching have provided introductions via short experiments aimed at undergraduates and even high school students [10,11,12,13,14]. Such “nanoprojects” for students strive to illustrate key concepts of nanoscience, often in a visually appealing manner, utilizing inexpensive and readily available equipment. Key, evident trends are continuously minimizing health and safety risks for relatively inexperienced students and the increased use of “green” chemistry [15,16,17,18].

In this review we highlight the interdisciplinary educational opportunities offered by nanoporous silicon, a nanomaterial inadvertently first created in the 1950′s [19], but whose diverse attributes only emerged over the last three decades. Its educational potential for introducing the multidisciplinary nature of NST was first utilized in a university undergraduate-level course twenty years ago [20] but until recently has been restricted by significant health and safety issues associated with electrochemical synthesis (see Section 2.1). The emergence of much safer, accessible, and inexpensive means of fabrication (see Section 2.3), many further new uses (see Section 4.1) and other advances, suggests it is now a timely moment to promote its potential educational uses in more detail. Table 1 lists seven established key concepts of NST and how these can be linked to this nanomaterial.

We first provide a brief background to the nanomaterial and its parent semiconductor, then cover how synthesis options have now expanded to include safer and more accessible routes, highlight two of the tunable properties of nanoporous silicon that have given rise to substantial interest in academia and industry, and then summarize application areas. We finally discuss some attributes of nanoporous silicon with regard medical safety and environmental impact and conclude by giving some examples of past and future teaching projects. This review emphasizes the range of educational opportunities across NST and green entrepreneurship afforded with studies of this material.

### 1.2. Silicon and Nanoporous Silicon

Semiconducting silicon is now synonymous with both electronics and solar power: it dominates both industries with greater than 90% of their global markets. This has arisen not only due to its combination of relevant properties but also because of its abundance, nontoxic nature and relatively low cost compared to other semiconductors. The performance of electronic circuitry embedded in silicon chips has continuously improved, whilst costs have plummeted so that over 5 billion people can now own portable computers (mobile smart phones). The cost of silicon-based solar panels has also plummeted, whilst efficiencies have climbed above 25%, close to their theoretical limit of ~30% [22]. Despite these remarkable achievements, more and more attention is also given to the environmental impact of silicon-based industries [23,24,25,26,27]. 

There are different routes to porosify silicon, but the most popular has been electrochemical etching of wafers discussed in Section 2. This technique now has a history of development over 60 years, and pore sizes can be controlled from about 2 nm width all the way to microns by choice of silicon resistivity, electrolyte, and etching conditions. Property tunability in porous silicon arises from void content, skeleton size and via impregnation to form nanocomposites (see Section 3). Nanoporous silicon is attracting increasing attention because it is a form of silicon and has demonstrated new and broader functionality across varied industries [28,29,30] as surveyed in Section 4.

Education via solid-state silicon technology has reflected the core properties and uses of the semiconductor, examples being bandgaps and electrical transport [31], microelectronics [32], solar cells [33,34], micro electro-mechanical systems [35], and photonics [36]. Target student disciplines have thus been primarily electrical engineering and solid-state physics. We propose here that nanoporous silicon properties to be discussed in Section 3 can broaden the educational reach of silicon technology to include chemistry, biology, biomedical, and environmental science students.

## 2. Porous-Silicon Synthesis—The Need for Sustainable Approaches

### 2.1. Nanostructuring via Electrochemistry

The preparation of porous silicon is readily achieved via anodization of single crystal silicon in a HF-based electrolyte [37,38]. Depending on a number of experimental parameters (wafer resistivity/dopant type, magnitude/duration of bias, electrolyte composition, illumination, etc.), porous films can be constructed that can be broadly classified with regard to porosity according to IUPAC designation as micro (<2 nm), meso (2–50 nm), or macroporous (>50 nm) [39]. Etch duration can dictate film thickness, and such films can be detached (with a suitable burst of current) from the bulk substrate as free-standing membranes. If desired, such membranes can be mechanically ground and sieved into micro/nanoparticles of a target size range. Other more sophisticated pSi particle morphologies can be produced via periodic modulation of current density [40] or in combination with lithographic processes [41].

While the experimental requirements for pSi fabrication are straightforward (on a modest scale), the need for toxic HF use as an electrolyte discourages widespread adoption in an undergraduate laboratory instructional format. Thus, alternatives must be embraced, and opportunities for ecofriendly, green chemical routes are of great appeal in influencing suitable choices. For proper context, we next briefly outline the core principles of green chemistry and the needs posed by existing silicon processing. 

### 2.2. Principles of Green Chemistry

The essence of green chemistry is perhaps best articulated in a recent Inorganic Chemistry textbook: “Sustainable development has become the cultural, scientific, and technical imperative of the twenty-first century” [42]. There are twelve principles associated with Green Chemistry that are broadly embraced as a consequence (Table 2). 

In particular regard to porous-silicon fabrication, it is useful to break down the overall process into two categories and recognize the demands of each in light of these principles: (1) elemental silicon formation and (2) subsequent creation of nanostructured/nanoporous silicon from this solid Si.

For electronic grade Si, step (1) typically entails initial formation of solid “metallurgical grade” silicon, currently manufactured at scale by reduction of quartz with carbon at 1700 °C in blast furnaces, a process with not only extreme thermal requirements but also in copious formation of CO_2_ as well. Given the fact that production of 1 ton of Si requires 12 MWh of electricity consumed, researchers are striving to find less-energy-consuming routes [43]. That silicon then has to be purified through a careful recrystallization process to incredible levels (>99.999%) to become solar or electronic “grades”, ultimately (after a series of dicing, polishing, and associated steps) in the form of wafers. It is this material that is subsequently anodized (Section 2.1 above). 

### 2.3. Biogenic Porous Silicon

The recognition that silicon, in the form of SiO_2_, can accumulate in selected plants from the surrounding soil has led to multiple approaches to the formation of porous Si particles utilizing these plants as silicon precursors. From a green chemistry perspective, several principles are endorsed: less hazardous chemical syntheses; safer solvents; design for energy efficiency; use of renewable feedstocks; pollution prevention; and inherently safer chemistry. Fundamentally, we are embracing a synthetic route to forming elemental Si in a nanostructured/nanoporous form in a single step.

Some of the reported approaches rely on transformation of structurally well-defined silica motifs, such as diatoms, to produce elegant nano/microcrystalline Si platforms [44]. In our collaborative research we have emphasized the use of relatively abundant plant sources that readily scale up, with practical implications for ultimate commercialization. These choices include tabasheer (a silica-rich extract present in bamboo), rice husk, and horsetail (Equestium). Our groups’ collaboration [45,46,47,48] have demonstrated that pSi derived from magnesiothermic reduction of these plant sources can produce nanostructures for drug delivery. Other research groups [49,50,51,52,53,54] have focused on anode material for lithium batteries. Both application areas have generated industrial activity as well as academic research (see Section 4.1 and Section 6).

In any case, porosification/nanostructuring of the silicon is achieved during the silica reduction step, typically via magnesiothermic reduction in the 550–650 °C range:

SiO_2_ (s) + 2 Mg (g) → Si (s) + 2 MgO (s)

**Figure 1:**
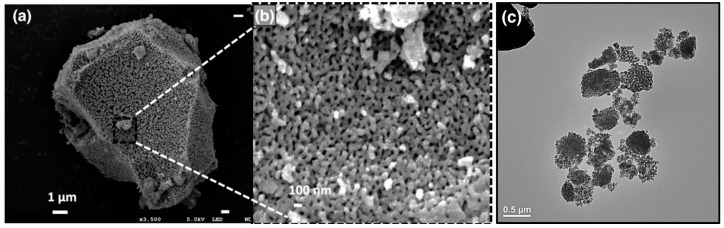
Electron microscopy images of plant-derived pSi: (**a**) field emission SEM image of a typical pSi microparticle derived from Tabasheer (scale bar = 1 μm); (**b**) magnified image of (a) showing surface pores in the mesoscale range (scale bar = 100 nm); and (**c**) TEM image of a pSi nanoparticle derived from ball-milled Tabasheer (scale bar = 500 nm).

While silica source particle size and morphology influence surface area and nanoscale morphology of the corresponding pSi product, the magnitude of the reaction enthalpy for this reduction step mandates a thermal moderator (e.g., inert salt such as NaCl) to suppress fusion of the pSi particles. Sizes in the micron range are readily obtained, with mesopores visibly evident by electron microscopy (Figure 1a), and proper milling of the silica source such as Tabasheer can reduce this particle size for the corresponding pSi product into the ~10^2^ nm regime (Figure 1c).

## 3. Porous Silicon Properties—The Need for Novel Functionality and Tunability

### 3.1. Luminescent Silicon and Nanomaterial Physics

All materials have their limits and for bulk silicon, one limit has been its exclusion from optoelectronics, due to its inability to emit light efficiently. This may change because we now know nanostructured silicon can luminesce very efficiently through quantum confinement effects [55]. However, the jury is still out regarding its future impact in optoelectronics, because what is currently demonstrated is very efficient photoluminescence (PL) but not electroluminescence (EL). Whilst PL efficiencies as high as 60–70% have now been achieved, the highest EL efficiencies are only around 10%.

**Figure 2:**
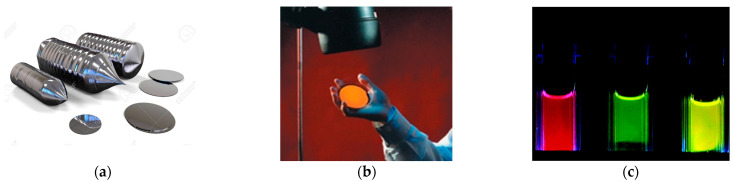
Conversion of solid silicon (**a**) to an orange photoluminescent nanoporous-silicon layer (**b**,**c**) shift in pSi emission from red → green → yellow as a consequence of reduced Si skeleton size via photocatalyzed dissolution of Si in HF as a function of time (Figure 2c adapted in part from [60] with permission from the American Chemical Society).

Figure 2 shows the size-tunable visible photoluminescence that students can see under UV illumination, from electrochemically etched nanoporous silicon. The morphology and precise size of the porous-silicon nanostructures emitting at specific wavelengths has been a controversial topic [55,56,57,58,59,60]. A detailed recent study [61] found an ensemble of 5 nm diameter isolated nanocrystals gave 1.4 eV emission (infrared), 3 nm gave 1.65 eV emission (red), and 2 nm diameter gave 2.0 eV output (yellow). By classroom discussions of selected papers, students can appreciate the significant technical challenges of nanostructure characterization and metrology (NST concept 3 in Table 1 and Photoluminescence Property in Table 3). They can also be introduced to quantum confinement effects and the origin of size-dependent properties (NST concept 4 in Table 1) via effective mass theory. 

**Figure 3:**
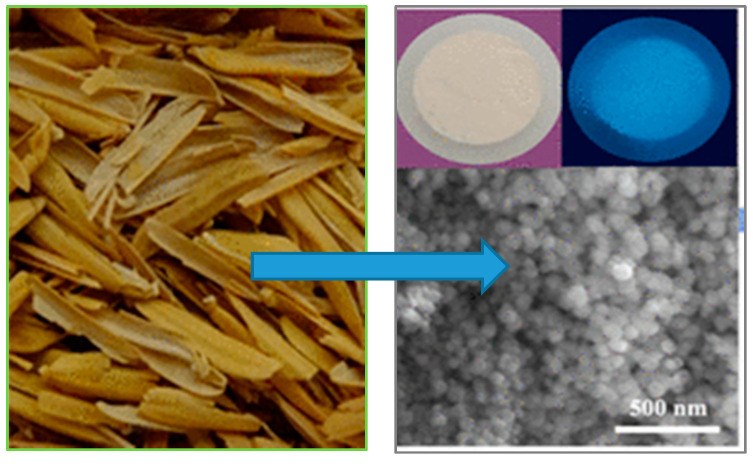
Conversion of rice husk to blue photoluminescent silica and carbon-based powders (Adapted from [64] with permission of the American Chemical Society).

A similar striking visual effect was first demonstrated with CdSe nanocrystals and led to a series of educational laboratory projects based on chemical synthesis and optical properties [10,11,62]. Luminescent Si nanocrystals have two clear advantages here (health and safety plus environmental) compared to highly toxic cadmium usage, provided we can avoid handling toxic hydrofluoric acid, as was discussed in Section 2. Currently, reported biogenic silicon and silica nanostructures have primarily exhibited blue-green luminescence [63,64,65]. An example is shown in Figure 3, where rice husks were calcined to a white powder that emitted blue light under UV. This luminescence probably arises from carbon contamination [55]. Further technical progress with improved size control, purity, and surface passivation is needed with biogenic porous silicon before high efficiency nanocrystal luminescence can be demonstrated in a classroom setting. One undergraduate experiment might thus be the comparison of pre-prepared luminescent silicon wafers with luminescent biogenic silicon that the students prepare from plants they have selected. In an existing 11-week 2nd year laboratory project at the University of Birmingham, students already choose varied plant extracts that they wish to use to create metal nanoparticles from metal–salt solutions. Here UV/vis spectroscopy is used to investigate plasmonic optical absorption, rather than efficient luminescence. The proposed project on luminescent nanoporous silicon would educate with regard six of the seven core NST concepts of table (concepts 1–5 and 7 in Table 1).

### 3.2. Biodegradable Silicon and Biomaterial Science

**Figure 4:**
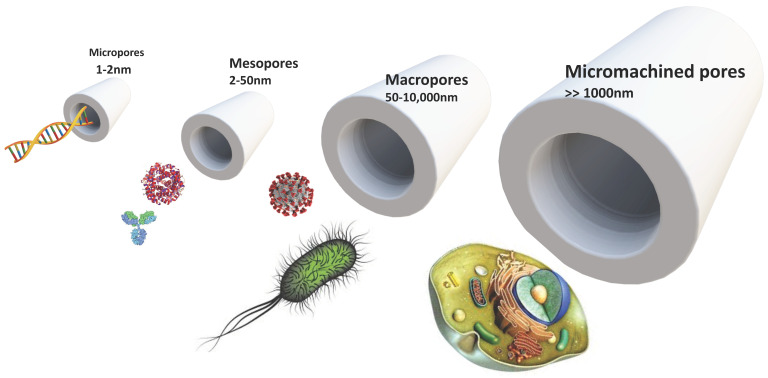
Pores and biological building blocks of varying size. Those chosen are DNA, antibodies, globular proteins, *E. coli*, coronavirus, and mammalian cells. Pore dimensions refer to diameters.

There have been some spectacularly successful medical devices, like the pacemaker, that rely on silicon-based VLSI circuitry. However, other materials have always been used, like biocompatible titanium, to interface those electronic devices with the body. Silicon itself was not considered a “biomaterial”. The continuing investigations into nanoporous-silicon bioactivity [66,67,68,69,70,71] and biodegradability [66,72,73,74] are allowing nanoporous-silicon researchers to engage with the biomaterial science and medical communities. Students of this material thus have the opportunity to learn about multiple scientific disciplines via “nanomedicine” [75]: biomaterials science [76] as well as the bench to bedside biocompatibility testing process: in vitro cell culture, in vivo animal models, and clinical trials under regulatory processes [77]. The extremely well-developed techniques for sculpting silicon into complex shapes over a wide range of length scales also renders it a powerful educational tool for biomaterial science. Figure 4 shows schematically how the different classes (sizes) of pores introduced in Section 1.1 are linked to biological entities of widely varying size. Micropores can be utilized in controlling DNA transport; mesopores in antibody and protein delivery; macropores are of comparable size to viruses and bacteria; but cellular in-growth for vascular tissue interfacing requires pores of hundreds of microns diameter. By comparing the size domains of different silicon processing tools (lithography, micromachining, and electrochemical etching) with biological building blocks (vascular tissue, cells, bacteria, viruses, proteins, and biomolecules) they can appreciate how topography as well as biochemistry influences material interactions with natural systems. This is at the core of NST concept 1 in Table 1. There is also an important trend towards “hierarchical” nanostructuring of synthetic biomaterials [78,79,80] utilizing more than one processing tool, that try to replicate the hierarchical porosity of some natural materials. Biodegradable scaffolds for tissue regeneration are clearly a good example of this, where the ability to control topography from nanometers to millimeters is required. The hierarchical structuring of silicon is being developed for numerous application areas shown in Table 4 [81,82,83]. In this regard, using a hierarchical biogenic silica template (Section 2.3) rather than multiple processing tools is particularly attractive, provided its dimensionality and topography are already optimized by nature for the specific use in mind.

The most advanced medical applications are brachytherapy, ocular drug delivery, and theranostics (see Table 4). The latter application utilizes both the luminescent and biodegradability properties. All therapies require biocompatibility, and, in most cases, biodegradability is a big advantage. Undergraduates can investigate the degradability of nanoporous silicon in various environments ranging from water of varying pH to simulated body fluids to seawater. Simple recipes for simulating physiological and environmental media are readily available. Students can, for example, study how biodegradability kinetics of biogenic porous silicon or electrochemically etched films vary with surface area, pore size, or environment pH. Corrosion can be monitored optically, chemically, gravimetrically, or by microscopy. This proposed project would educate with regard all seven core NST concepts of Table 1.

### 3.3. Silicon with Tunable Properties

**Figure 5:**
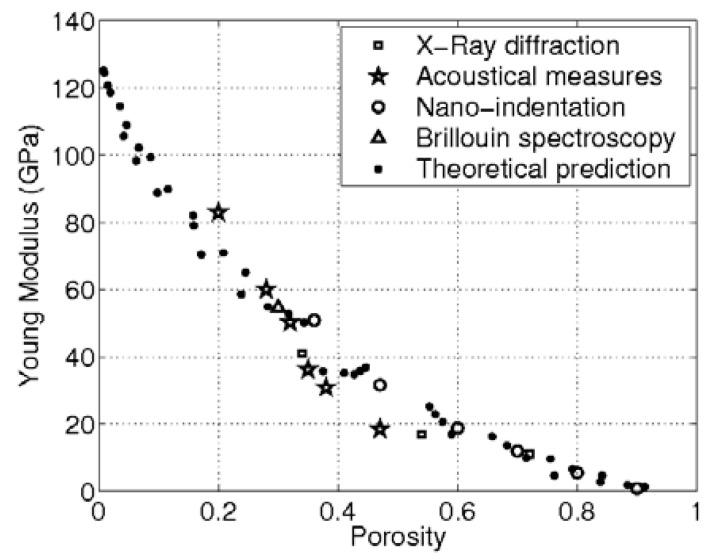
Tunable mechanical property of porous silicon: experimental data and theoretical modeling [85].

**Figure 6:**
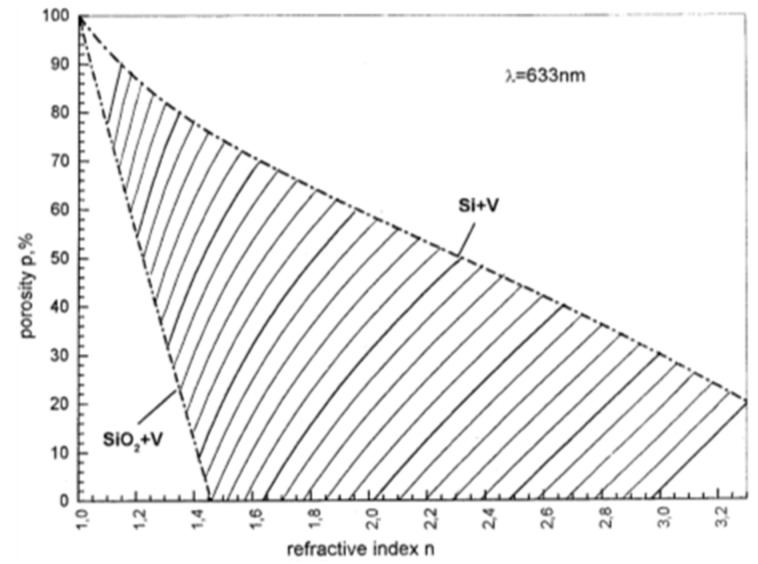
Tunable optical property of porous silicon: theoretical modeling of refractive index dependence on both porosity and level of oxidation. Adapted from [86] with permission of Elsevier.

Introducing nanoporosity into solid silicon can radically change its properties in a tunable manner [84]. Table 3 provides quantitative data for 10 properties of silicon. Densities in the range 0.12 to 1.9 g/cm^3^ correspond to porosities of 95 to 20%. Crystalline silicon with a Young’s modulus of 160 GPa is a very stiff (and brittle) material, but that stiffness can be lowered by up to two orders of magnitude by high porosity. Figure 5 emphasizes the tunability of this mechanical property. By contrast, the optical bandgap can be doubled or even tripled in size via quantum confinement effects. Another optical property, refractive index is also highly tunable (lowered) by porosity as shown in Figure 6. This has been utilized to make various micro-optical devices such as diffraction gratings, mirrors, waveguides, and cavities (see Table 4).

Electrical resistivity is one of the few highly tunable properties of bulk silicon. Impurity doping in ultrapure single crystals can tune resistivity precisely over five orders of magnitude, and indeed, this underpins its widespread use in electronics. Resistivity values for nanoporous silicon span nine orders of magnitude, and tunability here is dominated by skeleton dimensionality and crystallinity combined with surface chemistry, no longer by dopant concentration. Bulk-crystalline silicon is quite a good thermal conductor (150 Wm^−1^ K^−1^), but high-porosity silicon becomes a thermal insulator with conductivity values as low as 0.03 Wm^−1^ K^−1^. For comparison, the thermal conductivity of air at 0 °C is 0.024 Wm^−1^ K^−1^. Hydrophilicity (wetting by water) of bulk-silicon surfaces is tunable by surface chemistry. A wider tunability is achieved with nanoporous silicon where surface chemistry and physical morphology effects can be combined to promote super hydrophobicity (ultrahigh contact angles >150°) or superhydrophilicity (ultralow contact angles <5°). 

Bulk silicon does not dissolve in body fluids over useful timescales, but nanoporous silicon does and hence has biodegradability as a key property for various medical uses (Section 3.2). Bulk silicon has very inefficient luminescence processes in the near infrared. Nanoporous silicon exhibits efficient photoluminescence in the visible range due to quantum confinement effects on its band structure, and that luminescence is wavelength tunable right across the visible from deep red to blue (orange-yellow emission is shown in Figure 2). Finally, nanoporous films and microparticles loaded with oxidants can have explosive properties tunable by surface area. Indeed, impregnating the pores of the material provides a further route to both novel and tunable functionality [87].

## 4. Porous Silicon Uses—The Need for Product Safety, Reliability and Scalability

### 4.1. Diverse Applications, Their Associated Industries and Educational Topics

Table 4 groups together 20 different demonstrated functions of nanoporous silicon that span many industries and scientific disciplines. At least 45 related educational topics are represented. This provides students and teachers with a wide choice of specialization that can reflect interest, core discipline, breadth of expertise, and facilities available.

### 4.2. Medical Safety and Environmental Impact

The medical uses of nanoporous silicon (Table 4) can be used to illustrate the complex and lengthy process via which medical safety is assessed for new materials and new technologies. Nanoporous silicon has now progressed from preliminary in vitro tests in 1995 through numerous in vivo animal model testing to clinical assessment since 2004 [108]. Physics and chemistry students can learn about biomedical engineering and biomaterials. Biomaterial evolution is moving away from “bioinert” materials like stainless steel to biodegradable materials and “bioactive” implantable devices that help the body heal itself. There is now a medical toolbox of biodegradable polymers, ceramics, and metals. Even fully biodegradable electronic implant systems (power supplies, circuitry, and packaging) are under development. Medically biodegradable nanoporous silicon therefore represents this new class of medical materials and is the first biodegradable semiconductor.

Environmental impact of materials is particularly important for those that are manufactured at very high volumes. The marine environmental crisis of microplastics unfortunately demonstrates this [109]. Once again, there has been a growing interest in designing new materials of limited durability, helping the environment heal itself from our misguided actions. However environmental conditions are highly variable, very different from the homeostasis within the human body. Our environment can have widely varying temperatures, humidity, sunlight, oxygen levels, and types and concentrations of microorganisms. Environmentally degradable plastics that are simultaneously stable during storage and widespread use is a much greater challenge than medically biodegradable plastics [110].

**Figure 7:**
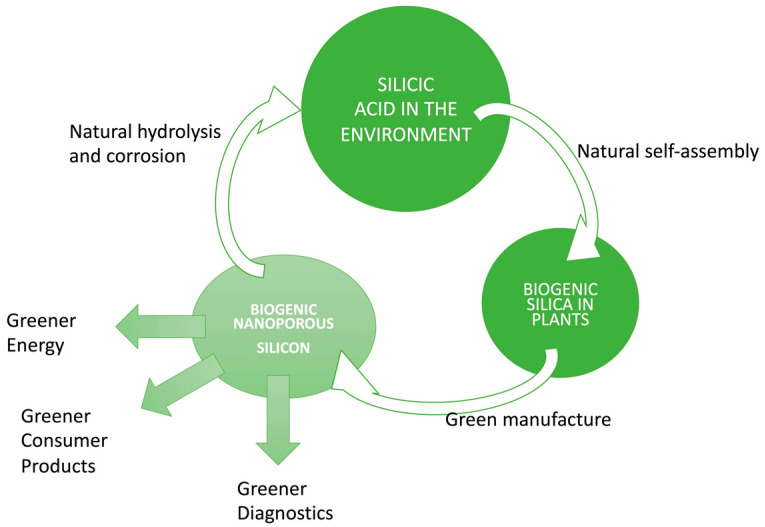
A silicic acid cycle—through soil, plants, and silica, to silicon nanostructures and back to soil.

Orthosilicic acid is the natural molecular form of silicon in our soil, rain, rivers, and seas. It is estimated that every year, trillions of tons of this molecule is cycled around our ecosystem. Plants alone uptake billions of tons every year [111]. Not only humans, but all of our wildlife, from insects to elephants, are accustomed to silicic-acid metabolism. Some animals, like the giant panda, even thrive on very high intake of biogenic silica via bamboo. Human production of significant amounts of biogenic silica and silicon is hence likely to have minimal effect on the environment and wildlife, provided the nanostructures degenerate into silicic acid. Figure 7 illustrates potential silicic acid cycling through soil, plants, and silica, to silicon nanostructures and back to soil. The key step in environmental impact is the conversion of nanoporous silica to nanoporous silicon as this requires elevated temperature manufacture and use of reducing agents like magnesium, calcium, or aluminum (Section 2.3). The conversion of nanoporous silicon to silicic acid in soil and oceans is likely to occur on highly variable and much longer timescales than have been quantified for physiological conditions. Nonetheless, a range of much more durable silicon compounds are already in use as “silicon fertilizers” for specific crops [112].

## 5. Case Studies and Proposals on Education via Nanoporous Silicon

### 5.1. Undergraduate NanoPhysics Lecture Course at University of Birmingham, UK 

**Figure 8:**
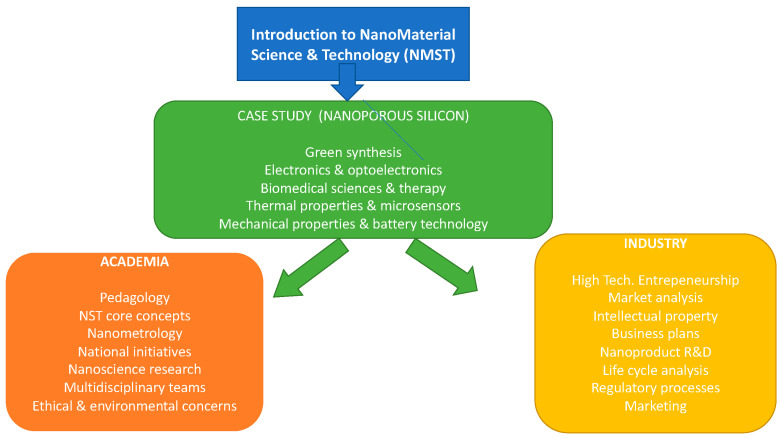
Lecture course introducing NMST and various aspects of academic research and industrial R&D in nanomaterials.

A lecture course was given for many years (from 2007 to 2014) to 3rd and 4th year physics undergraduates, using nanoporous silicon as a case study, as part of a module on nanophysics. The lectures were based on the flow diagram shown in Figure 8. The tuning of optical, thermal, and mechanical properties of silicon via nanostructuring (Section 3.3) were introduced. The novel properties of nanoporous silicon and thereby broadened functionality (Section 3.1 and Section 3.2) were also covered. Student discussion on the differences between academic and industrial research was stimulated. Multidisciplinary scientific topics, issues, and language underpinned much of the lectures. Examination questions highlighted physical-science concepts but included basic knowledge gained from the lectures in noncore disciplines. On the entrepreneurship side (Section 5.3), students were able to see examples of market analysis reports, invention reports, patents, and business plans. Microelectronic implants for therapy or augmentation and microelectronic tagging of humans (as opposed to animal pets) were used to introduce ethical issues. 

### 5.2. Undergraduate Research Activities Featuring Plant-Derived pSi at Texas Christian University, US

The relatively straightforward nature of the magnesiothermic reduction process has given rise to multiple undergraduate research projects on this topic in the Coffer Laboratories. We illustrate the opportunities afforded using this approach with two examples: (1) control of plant-derived pSi particle morphology by identity of plant component for a single plant type and (2) use of plant-derived pSi to serve as an ecofriendly drug carrier for sustained release of naturally occurring therapeutics such as allicin in garlic. 

**Figure 9:**
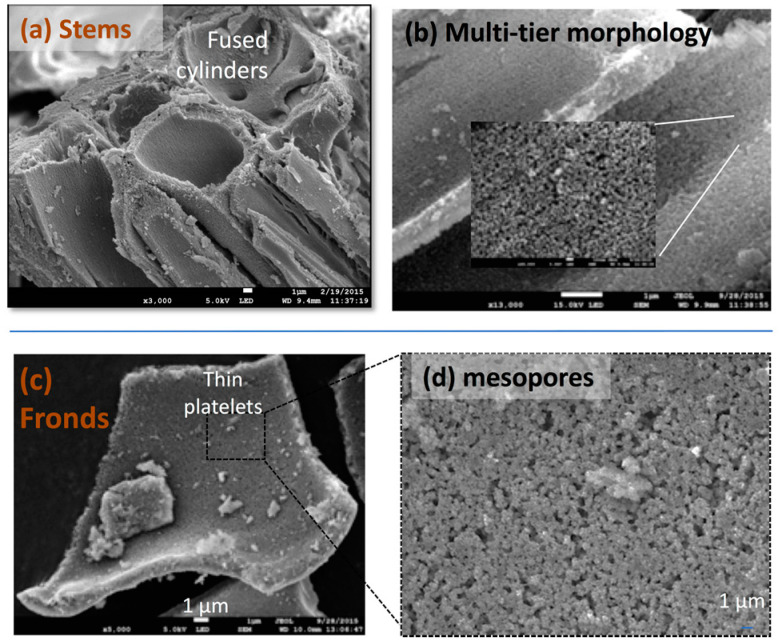
SEM images of pSi derived from *Equisteum Telematia*. The stem component of the plant produces pSi structures that are composed of fused cylinders (**a**,**b**) with mesoporous sidewalls, while the fronds generate rather thin platelets (**c**) that also possess a mesoporous morphology (**d**).

For the former, in 2013–2015, Sabrie Howell (SH), undergraduate TCU student, undertook a research project in the Coffer Group regarding the following hypothesis: does plant component structure in silicon-accumulator plants, such as horsetail, influence morphology in the resultant pSi product? This work was carried out in collaboration with LTC as the pSi from *Equisteum Telematia* was collected from a site near Malvern, UK and initially processed there. Fronds were carefully separated from the stems of the plant by the UK group and sent to TCU for calcination and subsequent magnesiothermic reduction by SH. Subsequent characterization by electron microscopy revealed a different morphology for the stem-derived pSi than for the frond-derived product (Figure 9) [113]. 

The drug-loading capacity and corresponding release kinetics of each type of pSi particle are anticipated to be quite different and currently under investigation. 

In another study, mesoporous silicon (pSi) derived from the silicon-accumulator plant Tabasheer (*Bambuseae*) was demonstrated by then undergraduate Nguyen Le to serve as a matrix for carrying and stabilizing naturally active, but otherwise metastable, therapeutic agents [47]. Particularly, garlic oil containing phytochemicals (namely, allicin) that are capable of inhibiting *Staphylococcus aureus* (*S. aureus*) bacterial growth were incorporated into this Tabasheer-derived pSi. Thermogravimetric analysis (TGA) indicated that relatively high amounts of the extract (~50 wt %) loaded into pSi are possible by simple infiltration. Importantly, by assessing the antibacterial activity of the samples using a combination of agar-disk diffusion and turbidity assays against *S. aureus*, NL determined that biogenic porous silicon can be utilized to stabilize and enhance the therapeutic effects of garlic oil for up to 4 weeks when the samples were stored under refrigerated conditions (4 °C) and 1 week at room temperature (25 °C).

Critically, under ultraviolet (UV) light (365 nm) irradiation for 24 h intervals, plant-derived pSi was shown to have superior performance in protecting garlic extracts over porous silica (pSiO_2_) derived from the same plant feedstock or extract-only controls. The mechanism for this effect was also subsequently investigated.

### 5.3. Entrepreneurship in NST

Although scientific entrepreneurship and path to commercialization [114,115] clearly involves multiple steps that need to be clearly articulated to students, JC has, as a Faculty Fellow in Entrepreneurship (2010–2014) with the TCU Neeley School of Business focused on communicating the basic steps of the process to both undergraduate and graduate students in selected coursework.

From 2010 through 2014, this was achieved through the use of the Senior Seminar Course (CHEM 40091) as a mechanism for introduction of the basic concepts of technology development and transfer in the sciences. Taught in a modular context, objectives included:

- motivational factors for entrepreneurship in the sciences [116,117,118,119];
- the key role of idea generation [120,121];
- the evolving role of the business plan [122,123];
- intellectual property (IP) and protection [124];
- funding mechanisms [125,126,127,128,129];
- unique regulatory considerations associated with biotechnology [130,131].
- careers in bioscience [132].

Short-term assessment was achieved via brief survey instruments sampling core student knowledge in topics such as business plans, intellectual property, and funding sources for start-up companies, in a before/after context.

#### 5.3.1. Idea Generation and Translation

The discovery of bioactivity in mesoporous silicon, coupled with the diverse tuneability of its pore size, surface chemistry, and particle size (among other properties), directly led to the realization by one of the authors (LTC) that multiple commercial opportunities might be possible. Subsequent entrepreneurial activities by LTC led to the creation of extensive IP and the formation of multiple companies—PSiMedica Ltd. UK, PSiOncology Pte Singapore, AION Diagnostics Australia, PSiVida Ltd. USA, and Intrinsiq Materials Ltd. UK—whose core technologies centered on the above-highlighted properties of porous silicon. Other start-up companies using different inventions followed in later years (Spinnaker Biosciences USA; Pico Technologies Nano Materials Co Ltd., Korea; TruTag Technologies Inc, USA; AMMT, Germany; Well Healthcare Technologies, China; SiLiMiXT-SAS, France; NacoMed, Norway; E-magy BV, Holland; LeydenJar, Holland; XNRGI, USA; Apollon Solar, France; HPQ Silicon Resources Inc, Canada; LumiSands Inc., USA; and NanoMedical Systems Inc., USA). 

These activities, in turn, have served as excellent case histories for educating students as to basics of entrepreneurship. The starting point in any entrepreneurial activity of course is idea generation, and live presentations by individuals who can share their vision and enthusiasm that guided their efforts are often the most effective mechanism for communicating this process to students (some of whom possibly become entrepreneurial in the long term). In presentations to students in an upper-level seminar course (CHEM 40091) in the TCU Department of Chemistry and Biochemistry, LTC has described the challenges and processes of translation of the useful fundamental properties of pSi nanomaterials outlined above into commercial ventures. The vast majority of start-up companies do not succeed in the long term and the first attempts to commercialize nanoporous-silicon products illustrate this sobering reality. Medical applications also follow lengthy clinical assessment prior to extensive use and marketing. Assessment of student learning of the basics of scientific E-ship, as gauged by differences between pre- and post-course completion surveys noted an increased ability to identify the basic steps of scientific entrepreneurship (+60–90%) and how porous silicon exemplifies these steps on the path to commercialization (+100%).

#### 5.3.2. Porous Silicon IP as Patent Critiques

Intellectual property is having a massive impact on the industrial development of NST with huge numbers of patents creating a dense “patent thicket” that companies have to navigate a way through. Thousands of patents on NST are now filed globally every year [133,134,135,136]. The USPTO, for example, currently lists more than 500 granted patents on porous silicon. 

Intellectual property on nanoporous silicon, like the academic literature, also cover many high-tech scientific disciplines, as illustrated in Section 7. Here we have chosen 30 granted patent examples from the last 20 years. Numerous inventors at large multinational companies, start-up companies, government laboratories, technology transfer institutes, and universities are represented. Many massive companies are evident that we associate with state-of-the-art electronics, like Intel Corporation, Samsung, and Hewlett Packard, as are institutions and start-ups whose core interests are in the biomedical field. Regarding biomedical uses, students can learn how very broad claims on known materials and structures are patentable under “first medical use” patent-law rules.

Indeed, the patent portfolio generated by LTC for PSiMedica Ltd. UK has also been utilized by other institutions in the context of student-driven critiques of patent quality. Specifically, in the fall of 2005, electrical-engineering students at the Massachusetts Institute of Technology (MIT), USA, have utilized one of the pSiMedica Ltd. patents in the EE course 3.172: Patents and Inventions [137]. These students critiqued United States Patent 6,666,214 (awarded December 23, 2003). The primary independent claim of this patent describes the following: a method of implantation comprising the step of implanting an electronic device within a living human or animal body, wherein the device includes bioactive (i.e., mesoporous) silicon. From the perspective of these students, this patent is classified as “powerful” because the associated definition of “bioactive silicon” is also very broad: “A ‘biomaterial’ is a nonliving material used in a medical device which is intended to interact with biological systems. Such materials may be relatively ‘bioinert’, ‘biocompatible’, ‘bioactive’, or ‘resorbable’, depending on their biological response in vivo.” 

In their review, these students further explain the significance of narrowing this independent claim with the following dependent claim: “6. A method according to claim 1, wherein the bioactive silicon is polycrystalline silicon. More specifically: Bulk-crystalline silicon can be rendered porous by partial electrochemical dissolution in hydrofluoric acid-based solutions, as described in U.S. Pat. No. 5,348,618. This etching process generates a silicon structure that retains the crystallinity and the crystallographic orientation of the original bulk material. The porous silicon thus formed is a form of crystalline silicon.”

Interestingly, these students suggest that demonstration of the bioactive nature of this silicon-living organism interface has significant claims on other future methods of inserting silicon devices into the body.

### 5.4. Interdisciplinary NST Schools and Conferences

Another instructional mechanism in NST with a focus on nanostructured silicon is the Summer School for Silicon Nanotechnology (SSSiN), an immersive six-week workshop on the synthesis, properties, and applications of silicon-based nanomaterials at the University of California (UC), San Diego. Begun in 2003, offered on an annual basis, and affiliated with the Silicon Nanomaterials Research Laboratory of Prof. Michael Sailor, it integrates participants from a wide variety of backgrounds and skill levels: high school students, undergraduates, graduate students, post-docs, industry researchers, and university professors. At present it alsoserves aspart of theResearch Immersion in Materials Science & Engineering (RIMES)training program of the UC San DiegoNational Science Foundation (NSF) Materials Research Science and Engineering Center (MRSEC),a “bootcamp” for selected students at the beginning of their MS or PhD degrees and for MRSEC ResearchExperience forUndergraduates (REU) participants. Course elements include lectures, hands-on laboratory training, and a capstone “Discovery Project”—an independent research project implemented by a team of trainees under the mentorship of a current research group member [138].

With regard to targeted conferences addressing nanostructured silicon, a biannual international conference “Porous Semiconductor Science and Technology (PSST)” has also provided multidisciplinary education to students and researchers at multiple levels from 1998 to the present day. As with the aforementioned summer school in the US, undergraduate participation in this European conference is encouraged. The week-long international conference has a prelude set of informal tutorials for student attendees, each of 30 min duration, covering multiple scientific disciplines. The choice of disciplines reflects evolving research activity levels. In 2016, for example, tutorials covered electrochemistry, sensors and microsystems, medical therapies, acoustics, and energy technologies. Although the 2020 PSST Conference was postponed by the Covid-19 pandemic, this meeting has been rescheduled for October 2021 in Lido de Camaiore, Tuscany, Italy [139]. 

## 6. Conclusions

In this article, we have detailed how the properties and uses of nanoporous silicon provide useful educational tools for NST, especially with the emergence of green synthesis routes. Important topics covered are plant-based synthesis, size, and porosity-tunable properties, multidisciplinary uses, product commercialization in the biomedical field, environmental impact, societal impact, and entrepreneurship. Following a collaboration between the authors lasting more than 25 years, our lectures, undergraduate training, and research projects from the US and UK that utilized porous silicon have been summarized. Potential future projects are proposed where students will fabricate luminescent or biodegradable structures using green chemistry and natural plant feedstocks. A significant number of start-up companies that use nanoporous silicon have emerged, and their case histories and IP can be used to educate students in the basics of high-tech entrepreneurship and intellectual property.

## 7. Patents

USP 6,017,773. **2000.** Stabilizing process for porous silicon and resulting light-emitting device. P.M.Fauchet et al. **University of Rochester,** USA.

USP 6,130,748. **2000.** Chemical sensor based on porous silicon. Kruger et al. **Forschungszentrum Julich** GmbH, Germany.

USP 6,248,539. **2001.** Porous semiconductor-based optical interferometric sensor. M.R.Ghadiri et al. **Scripps Research Institute** and USCSD, USA.

USP 6,380,550. **2002.** Electroluminescent device comprising porous silicon. L.T. Canham et al. **UK Ministry of Defence**, UK.

USP 6,666,214. **2003.** Biomaterial. L.T.Canham. **pSiMedica Ltd.,** UK.

USP 6,939,728. **2005.** Method of fabricating silicon emitter with a low-porosity heavily doped contact layer. X. Sheng et al. **Hewlett Packard LP**, USA.

USP 7,101,772. **2006.** Means for forming SOI. T.W. Houston et al. **Texas Instruments Inc.,** USA.

USP 7,515,851. **2009.** Electron emitter, charger, and charging method. H. Hirakawa et al. **Sharp Kabushiki Kaisha,** Japan

WO 108479. **2009.** Porous-silicon quantum-dot photodetector. H.J. Hovel et al. **IBM Corp**., USA.

WO 038068. **2010.** Cosmetic formulations comprising porous silicon. L.T. Canham and T. Monga. **Intrinsiq Materials Global Ltd.,** Ireland.

WO 114126, **2011.** Mesoporous silicon. L.T. Canham and A. Loni. **pSiMedica Ltd.,** UK.

USP 7,897,181. **2011.** Method for photothermal therapy using porous silicon and near infrared radiation. C. Lee et al. **Inha-Industry Partnership Institute**, Korea.

USP 7,942,989. **2011.** Porous-silicon-based explosive. M.J. Sailor et al. **University of California** San Diego, USA.

USP 7,980,828. **2011.** Micro-electro-mechanical pump utilizing porous silicon. J.W. Lantz et al. **Sandia Corp,** USA.

EP 1919302. **2011.** Food comprising silicon. L.T. Canham. **Intrinsiq Materials Global Ltd.,** Ireland.

USP 8,097,236. **2012.** Devices and methods for the treatment of cancer. R. Aston and L.T. Canham. **pSiMedica Ltd.,** UK.

USP 8,453,929 **2013**. Producing a microtag identifier. T. Learmonth et al. **TruTag Technologies Inc.,** USA.

USP 8,506,530. **2013.** Microneedles to be placed in the skin for the transdermal application of pharmaceuticals. Laermer et al. **Robert Bosch** GmbH, Germany.

USP 8,540,862. **2013.** Method of forming laterally graded porous silicon optical filter through diffusion-limited etching and filter structure manufactured thereby. H.S.Jeon et al. **Samsung Electronics Co**. Ltd., Korea.

USP 8,709,355. **2014.** Microfluidic system incorporating a metal impregnated nanoporous material in a microfluidic pathway thereof. S. Chan et al. **Intel Corp**., USA.

USP 8,729,798. **2014.** Antireflective nanoporous silicon for efficient hydrogen production. J. Oh et al. **Alliance for Sustainable Energy**, USA.

USP 8,926,803. **2015.** Porous silicon electroetching system and method. D. Crafts et al. **Solexel Inc.,** USA.

USP 8,940,278. **2015.** Oral hygiene compositions. L.T. Canham, **Intrinsiq Materials Global Ltd**., Ireland.

USP 9,023,896. **2015.** Porous-silicon drug-eluting particles. P. Ashton et al. **pSivida Inc.,** USA.

USP 9,142,833. **2015.** Lithium-ion batteries based on nanoporous silicon. S.H. Tolbert et al. **University of California**, USA.

USP 9,466,662. **2016**. Energy-storage devices formed with porous silicon. D.S. Gardner et al. **Intel Corp.,** USA.

USP 9,511,998. **2016**. MEMS device having a getter. A. Samaro et al. **Robert Bosch** GmbH, Germany.

USP 10,090,513. **2018**. Method of forming silicon. L. Canham et al. **Nexeon Ltd.,** UK.

USP 10,204,836. **2019.** Porous-silicon relaxation medium for dislocation free CMOS devices. K. Cheng et al. **IBM Corp**., USA.

USP 10,825,954. **2020.** Porous-silicon light-emitting device and manufacturing method thereof. M. Morelli et al. **ST Microelectronics**, Italy.

## Figures and Tables

**Table 1:** Key concepts of nanomaterial science and technology (adapted from [9]) and their educational links to nanoporous silicon fabrication, properties, and applications (applications in italics were highlighted by the Delphi study of nanotechnology uses [21]).

NST Concept	Concept Description	KEY NST EDUCATIONAL TOPICS LINKED TO NANOPOROUS SILICON
1	Size and scale classification	Nanopores, mesopores, and micropores. Hierarchical porosity. Nanocrystals and nanoparticles. Quantum wires and quantum dots. Biological building blocks.
2	Nanofabrication methods	Top-down vs. bottom-up fabrication. Nanotools. Control over size and shape at the nanoscale. Scalability.
3	Characterization at the nanoscale	Amorphous vs. nanocrystalline physical states. Nanometrology. Surface and interfacial chemistry. Size and shape distributions.
4	Size-dependent properties	Properties tunable by porosity (surface area/volume) Properties tunable by silicon skeleton dimensionality Size-dependent toxicity. Medical bioactivity and biodegradability. Size-tunable luminescence.
5	Functionality	New functionality enabled by nanostructuring bulk silicon. Extended functionality via size-dependent properties.
6	Multidisciplinary nature	Understanding and exploitation dependent on combined expertise and knowledge from many scientific disciplines and industries (see Table 2). *Nanoelectronics. Nanomedicine. Solar Energy.*
7	Environmental & societal impact	Societal benefits. Sustainable synthesis. Material life cycle assessments. Risk assessments. Biodurability. Implantable electronics. *Nanobots.* Ethical issues.

**Table 2:** Principles of Green Chemistry.

1. Prevention
2. Atom Economy
3. Less Hazardous Chemical Syntheses
4. Designing Safer Chemicals
5. Safer Solvents and Auxiliaries
6. Design for Energy Efficiency
7. Use of Renewable Feedstocks
9. Catalysis
10. Design for Degradation
11. Real-time analysis for Pollution Prevention
12. Inherently Safer Chemistry for Accident Prevention

**Table 3:** Examples of widely tunable properties of nanoporous silicon compared to bulk crystalline silicon. (Adapted from [84]).

Property	Values for Crystalline Silicon	Ranges for Nanoporous Silicon
Density	2.33 g/cm^3^	0.12–1.9 g/cm^3^
Young’s Modulus	160 GPa	1–100 GPa
Optical bandgap	1.1 eV	1.1–3.2 eV
Refractive index	3.5	1.1–3.0
Electrical resistivity	0.01–1000 ohm cm	1000–10^12^ ohm cm
Thermal conductivity	150 Wm^−1^ K^−1^	0.03–20 Wm^−1^ K^−1^
Surface wettability (water contact angle)	5°–96°	0.5°–168°
Biodegradability kinetics	NA	Hours (nanoparticles) Days (microparticles)
Photoluminescence wavelength	1100 nm	400–1100 nm
Photoluminescence quantum efficiency	<0.001%	<32% (films) <60% (suspensions)
Burn rate	NA	1–1840 m/s

**Table 4:** Multifunctionality and uses of nanoporous silicon.

Key Property of Nanoporous Silicon	Function	Industry	EDUCATIONAL TOPICS	References
Enhanced chemical reactivity VLSI compatibility	Sacrificial material for lithographic patterning	ELECTRONICS	Photolithography Micromachining Microsystem designMEMS	[88] Muller (2010)
Low AC conductivity Low dielectric constant	RF electrical isolation	ELECTRONICS	RF semiconductor devices and circuitry.	[89] Gautier (2014)
Low thermal conductivity	Thermal isolation	ELECTRONICS	Microsensor design Heat transport	[90] Nassiopoulou (2014)
Photo- luminescence	Down-converter in white LEDs	OPTO- ELECTRONICS	Radiative processes Phosphors Color rendering	[91] Barillaro (2014)
Electro- luminescence	Active layer in LED	OPTO- ELECTRONICS	Quantum confinement Excitons LED technology	[92] Gelloz (2014)
Tunable Refractive index	Micro-optical devices	OPTICS	Micro-optics Photonic communication	[86] Astrova (2000)
High Surface area	Matrix for Sensing	DIAGNOSTIC	Sensor technologies Surface chemistry	[93] Harraz (2014) [94] Arshavsky- Graham (2019)
High surface area	Matrix for adsorption- photodesorption	DIAGNOSTIC	Mass spectrometry Metabolomics Forensics	[95] Wei (1999)
Neutron transmutable	Host for medical radioisotope	MEDICAL	Targeted cancer therapy (Brachytherapy)	[96] Zhang (2006)
Biodegradabilty	Drug delivery	MEDICAL	Pharmacology Biomaterial testing	[97] Anglin (2008)
Biodegradability Photo- luminescence	Theranostics	MEDICAL	In vivo imaging Contrast agents	[73] Park (2009)
Bioactivity	Bone growth stimulation	MEDICAL	Orthopaedics Tissue engineering	[98] Coffer (2014)
Nanoporosity	Nutrient protection	FOOD	Organoleptic assessment Food additives & GRAS status	[99] Shabir (2012) [100] Canham (2014)
UV absorption	Sunscreen	COSMETIC	Light scattering Sun Protection Factors	[101] Popov (2011) [102] Canham (2014)
Tunable hardness	Toothpaste Abrasive	CONSUMER CARE	Abrasive testing Consumer acceptance	[102] Canham (2014)
Lithiation capacity	Anode in Li ion battery	ENERGY CONVERSION	Battery technology Mechanical properties	[103] Li (2014)
Tunable Refractive index	Antireflection coating in silicon photovoltaics	ENERGY CONVERSION	Solar-cell technology Light reflection	[104] Dzhafarov (2018)
Tunable thermal conductivity	Conversion of temperature gradients to electrical power	ENERGY CONVERSION	Energy scavenging Thermoelectric materials	[105] Schierning (2014)
Low thermal conductivity	Conversion of electrical power to sound	ENERGY CONVERSION	Ultrasonics Phonon Confinement	[106] Shinoda (1999)
Rapid oxidation	Rapid conversion of chemical energy to heat	ENERGY CONVERSION	Pyrotechnics. Explosives. Propellants	[107] Du Plessis (2014)

## Data Availability

Representative examples of nanoquizzes and lecture notes noted above are available from the data repository maintained by the Mary Couts Burnett Library of Texas Christian University: https://doi.org/10.18776/tcu/data/43764.

## Supporting Information

Selected course content (nanoquizzes, lecture notes, and examination questions) on educational topics in green, nanoscale Si materials, and scientific e-ship is available from the authors upon request.
